# Identification of candidate biomarkers for GBM based on WGCNA

**DOI:** 10.1038/s41598-024-61515-3

**Published:** 2024-05-10

**Authors:** Qinghui Sun, Zheng Wang, Hao Xiu, Na He, Mingyu Liu, Li Yin

**Affiliations:** 1 NHC Key Laboratory of Tropical Disease Control, School of Tropical Medicine, Hainan Medical University, Haikou, 571199 Hainan China; 2 Biotechnology and Biochemistry Laboratory, Hainan Medical University, Haikou, 571199 Hainan China; 3 School of Stomatology, Hainan Medical University, Haikou, 571199 Hainan China

**Keywords:** Glioblastoma multiforme, Grade IV, WGCNA, Biomarker, Biomarkers, Tumour biomarkers

## Abstract

Glioblastoma multiforme (GBM), the most aggressive form of primary brain tumor, poses a considerable challenge in neuro-oncology. Despite advancements in therapeutic approaches, the prognosis for GBM patients remains bleak, primarily attributed to its inherent resistance to conventional treatments and a high recurrence rate. The primary goal of this study was to acquire molecular insights into GBM by constructing a gene co-expression network, aiming to identify and predict key genes and signaling pathways associated with this challenging condition. To investigate differentially expressed genes between various grades of Glioblastoma (GBM), we employed Weighted Gene Co-expression Network Analysis (WGCNA) methodology. Through this approach, we were able to identify modules with specific expression patterns in GBM. Next, genes from these modules were performed Gene Ontology (GO) and Kyoto Encyclopedia of Genes and Genomes (KEGG) enrichment analysis using ClusterProfiler package. Our findings revealed a negative correlation between biological processes associated with neuronal development and functioning and GBM. Conversely, the processes related to the cell cycle, glomerular development, and ECM-receptor interaction exhibited a positive correlation with GBM. Subsequently, hub genes, including SYP, TYROBP, and ANXA5, were identified. This study offers a comprehensive overview of the existing research landscape on GBM, underscoring the challenges encountered by clinicians and researchers in devising effective therapeutic strategies.

## Introduction

Glioblastoma multiforme (GBM), also known as grade IV astrocytoma with an extremely poor prognosis, is one of the most common primary malignant brain tumors in adults and one of the most malignant gliomas. GBM is characterized by strong invasiveness, a high recurrence rate, a low survival rate, and poor prognosis, with a median survival time of 12–15 months. The recurrence rate is faster after surgical resection because the resection fails to eradicate GBM cells completely. According to different research data, the 5-year survival rate of GBM is approximately 5–10%. Currently, the treatment methods for GBM mainly include surgical resection, radiotherapy, and chemotherapy. However, due to the high degree of invasiveness and drug resistance of GBM, these treatment methods are often difficult to completely cure patients. Therefore, researchers are working to find new treatment methods, such as targeted therapy and immunotherapy. The gene expression profile based on microarray has been applied to explore the pathogenesis of disease and proved to be a useful biomedical tool in the identification of biomarkers in many aspects^1,2^. Weighted gene co-expression network (WGCNA) can be employed to identify highly correlated genes, which can then be grouped into the same module. Furthermore, these modules may be associated with specific external traits^3^. As a statistical method used for studying gene co-expression networks WGCNA has already shown its advantage in screening hub genes in many aspects^4–6^. A WGCNA analysis conducted on the TCGA dataset found core genes related to poor prognosis in GBM, providing new clues for understanding GBM prognosis and the immune microenvironment^7,8^. Additionally, researchers have performed cell function experiments, in situ and subcutaneous xenograft tumor models to evaluate the impact and molecular mechanism of RPL22L1 on GBM. The results showed that RPL22L1 was significantly upregulated in GBM and was associated with poorer prognosis^9^.

Differentially expressed genes (DEGs) were derived from the expression profiles across various grades of glioma. Through the construction of interaction networks for these genes, we investigated the functions of DEGs associated with each glioma grade, aiming to uncover shared and distinct mechanisms of action. This approach facilitated the examination of common and grade-specific biological pathways, as well as molecular expression changes in different glioma grades. The study contributes to our understanding of the potential pathogenesis of glioma, offering a theoretical foundation for comprehending glioma at the molecular level and informing strategies for its prevention and control.

## Results

### Data processing

The expression matrix data for the series of 176 samples has been downloaded from NCBI. The four groups, namely normal, Grade 2, Grade 3, and Grade 4, comprise 23, 45, 31, and 77 samples, respectively. To comply with the input file requirements for WGCNA, separate expression and traits files were generated accordingly.

### Construction of weighted gene co-expression network and identification of modules related to external traits

**Figure 1:**
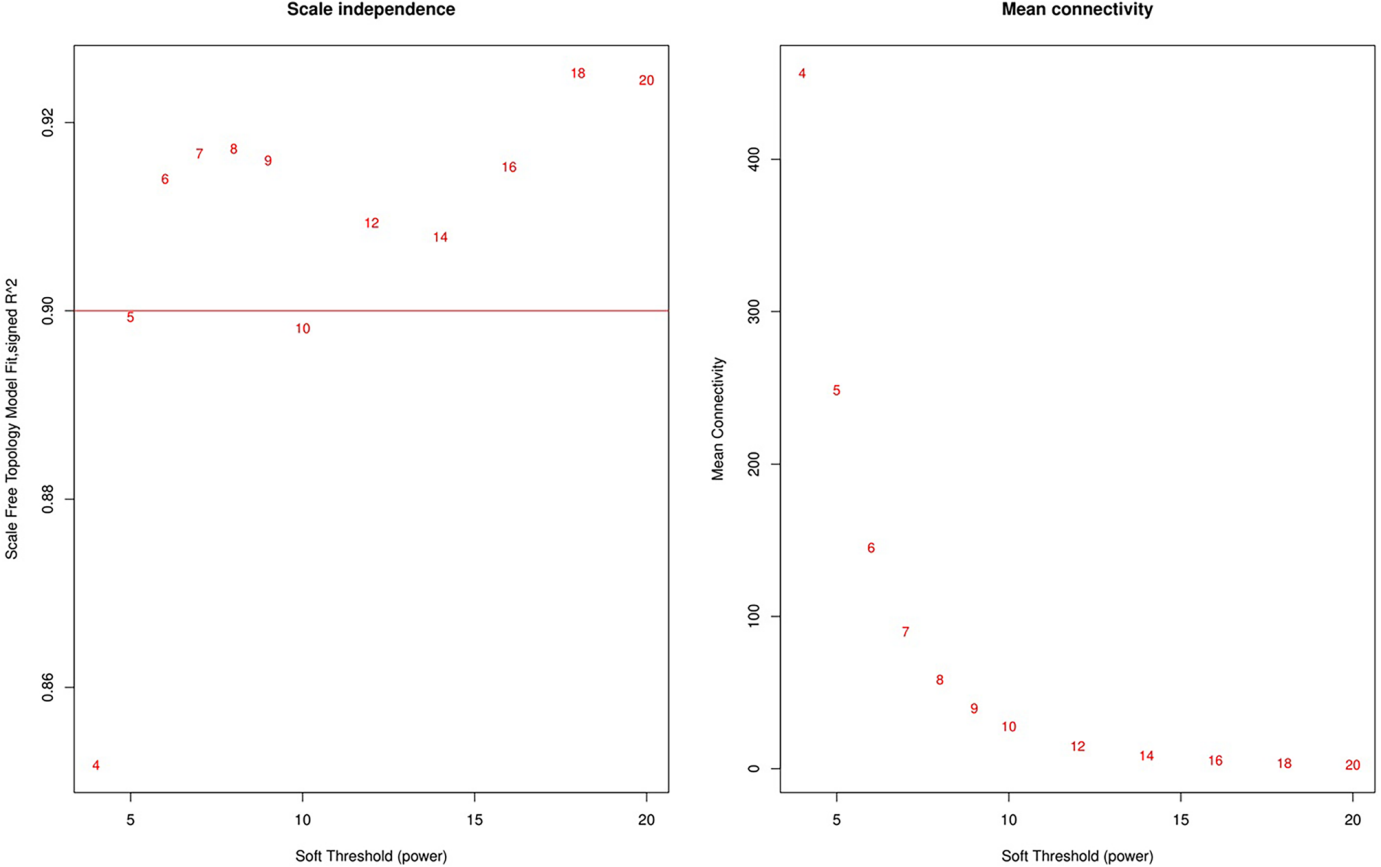
Network topology for different soft-thresholding powers.

The determination of the adjacency matrix for a scale-free topology network involved opting for a soft threshold power of 6, a decision made with the assistance of the pickSoftThreshold function in WGCNA, as depicted in Fig. 1. This specific value significantly influences the creation of modules and the allocation of genes within those modules.

**Figure 2:**
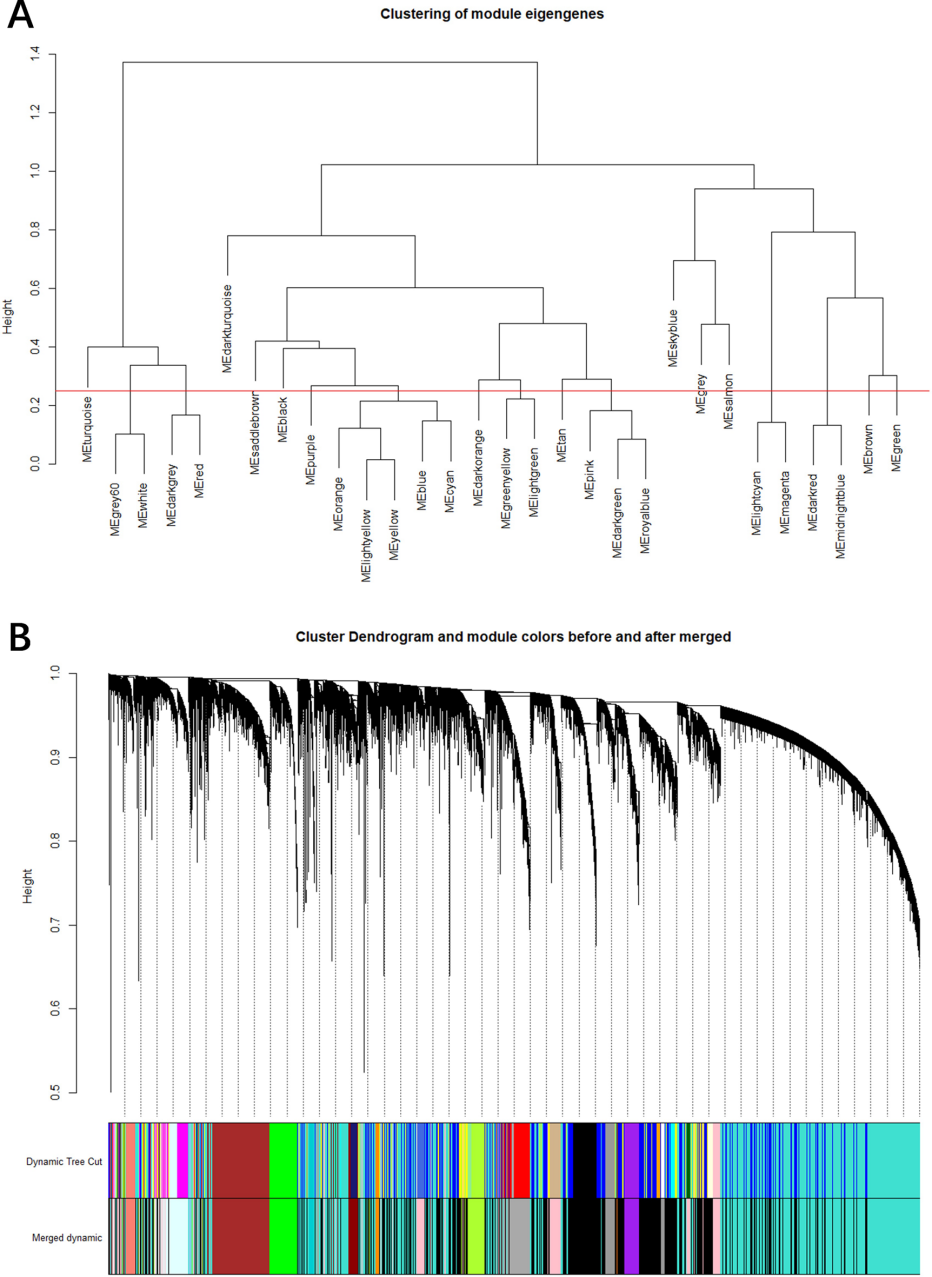
Modules identified by WGCNA. (**A**) Co-expression similarity of all modules based on hierarchical clustering of module eigengenes. The cut height of 0.2 is chosen to merge the similar modules. (**B**) The cluster dendrogram and color display of co-expression network modules for all genes. The short vertical line corresponded a gene and the branches corresponded the co-expressed genes.

This value corresponded to the minimum point nearest to the scale-free network. In our investigation, we utilized the dynamic tree cutting approach to discern genes demonstrating similar expression patterns along with their correlated biological processes and pathways. Following this, the modules will be subjected to clustering based on representative correlation features. In this scenario, a cut height of 0.2 has been chosen to merge modules with comparable characteristics (Fig. 2A).

The dendrogram was created through hierarchical clustering, where each short vertical line represented a gene, and the branches denoted co-expressed genes (Fig. 2B). It illustrated the cluster dendrogram and the module colors before and after merging. The upper part of the Fig. 2B with vertical black lines represents individual modules, with the height (tree cutting line) reflecting the degree of similarity between modules. The colored bars in the lower part of the Fig. 2B represent the colors assigned to each module, with the “Dynamic Tree Cut” row showing the initial module assignments and the “Merged dynamic” row showing the results after some modules have been merged.

### Correlation between modules and clinic traits

**Figure 3:**
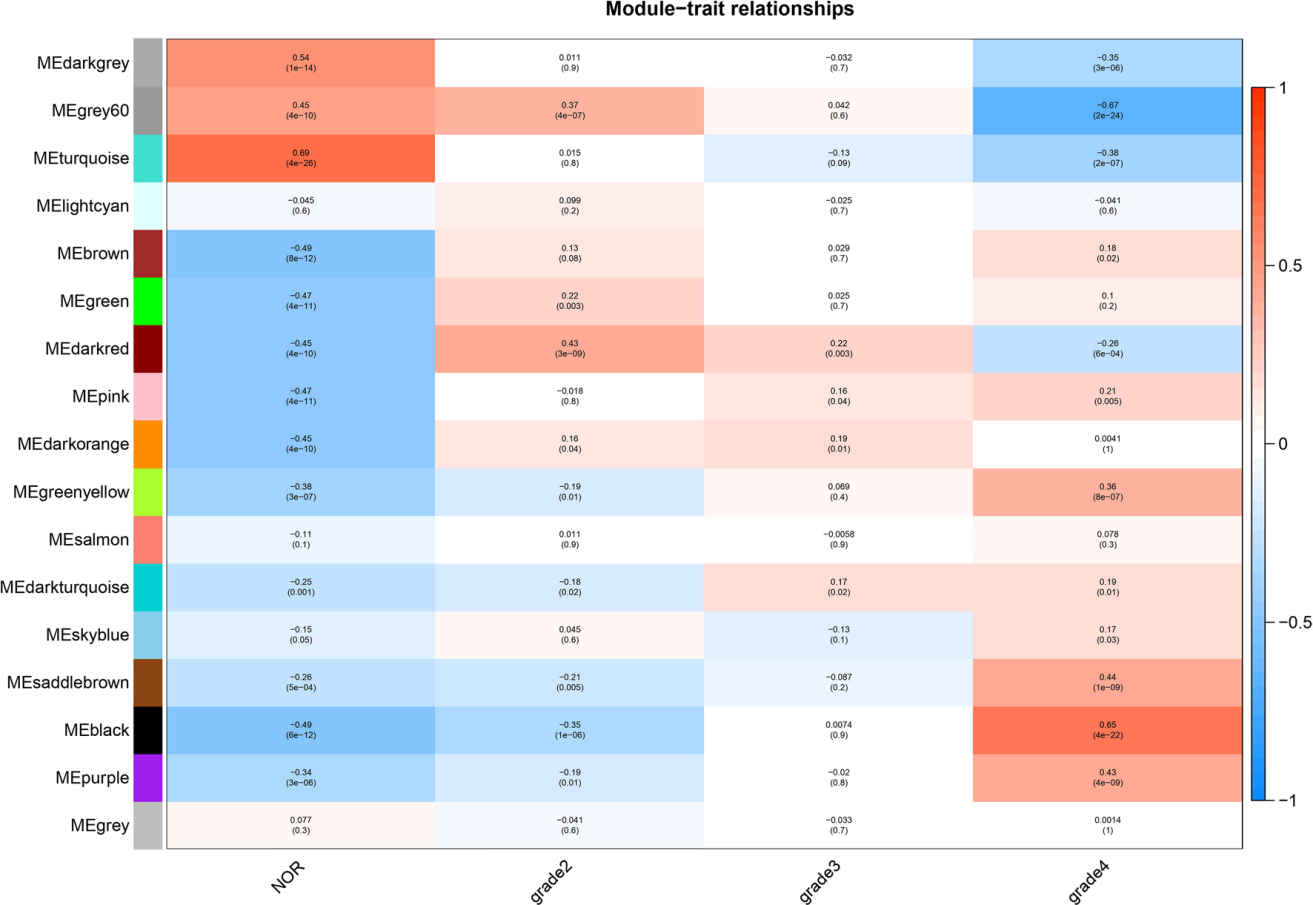
Correlation matrix of module eigengene values obtained from WGCNA. 17 modules were identified, and each module eigengene was tested for correlation with trait. Within each cell, upper values are correlation coefficients between module eigengene and the traits; lower values are the corresponding *p*-value.

Genes exhibiting similar expression patterns (co-expressed genes) were grouped into cohesive modules. Modules that showed a notable correlation with tumors and Grade 4 were specifically identified by evaluating the correlation between Module Eigengenes (MEs) and external traits, as illustrated in Fig. 3. Modules colored in saddlebrown, black, purple, and greenyellow were acknowledged for their positive association with Grade 4.

In particular, the black module has a p value of 4e−22. Darkgrey, grey60, turquoise and darkred modules were negatively associated with Grade4.

### Functional enrichment analysis

**Figure 4:**
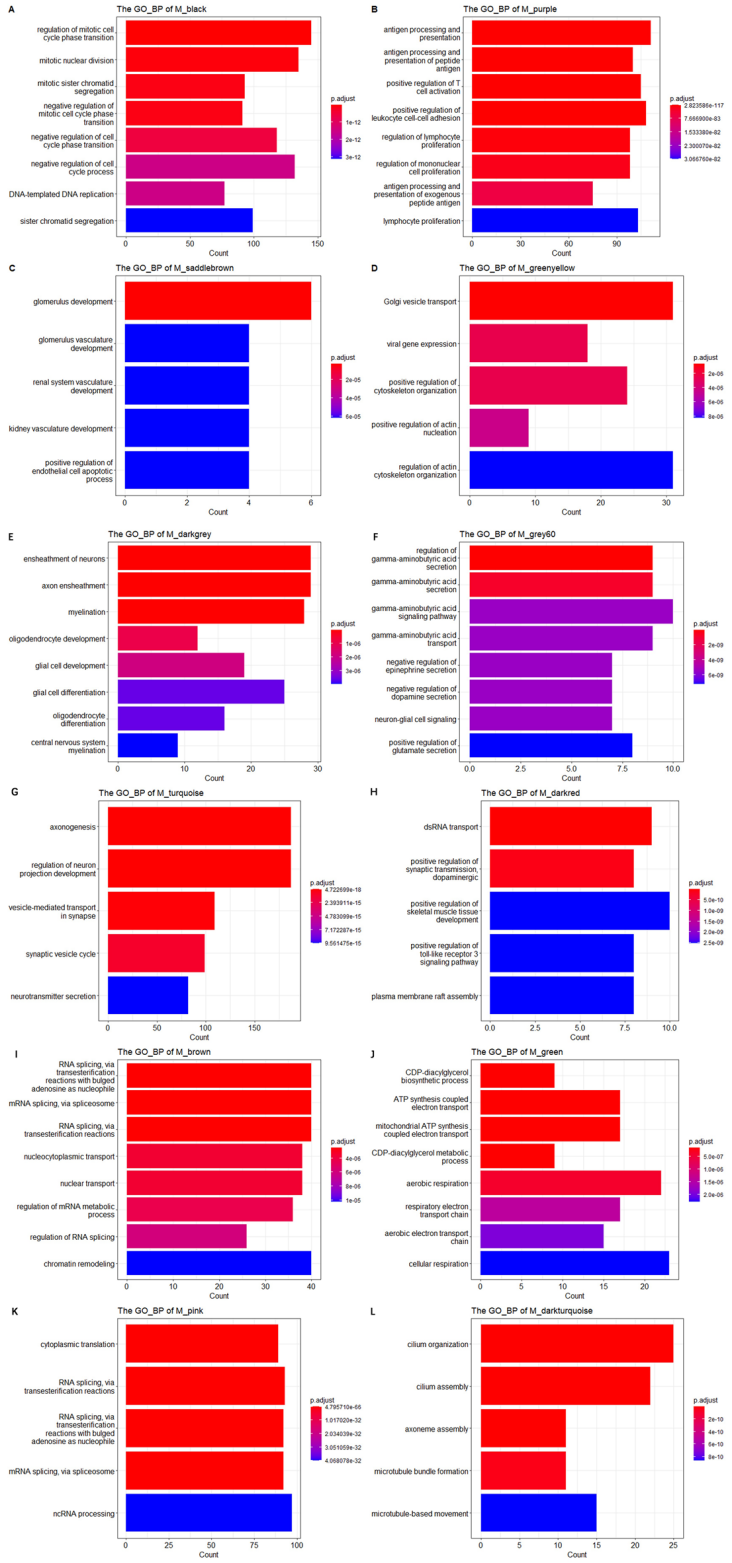
The enriched GO:BP terms of the genes for several modules. (**A**–**L**) Shows enriched GO:BP terms for black, purple, saddlebrown, greenyellow, darkgray, grey60, turquoise, darkred, brown, green, pink and darkturquoise modules respectively.

The genes in modules including Saddlebrown, black ,purple, greenyellow, Darkgrey, grey60, truquoise and darkred modules were selected to perform the GO:BP and KEGG enrichment analysis using ClusterProfiler package respectively, which were shown in Fig. 4.

**Figure 5:**
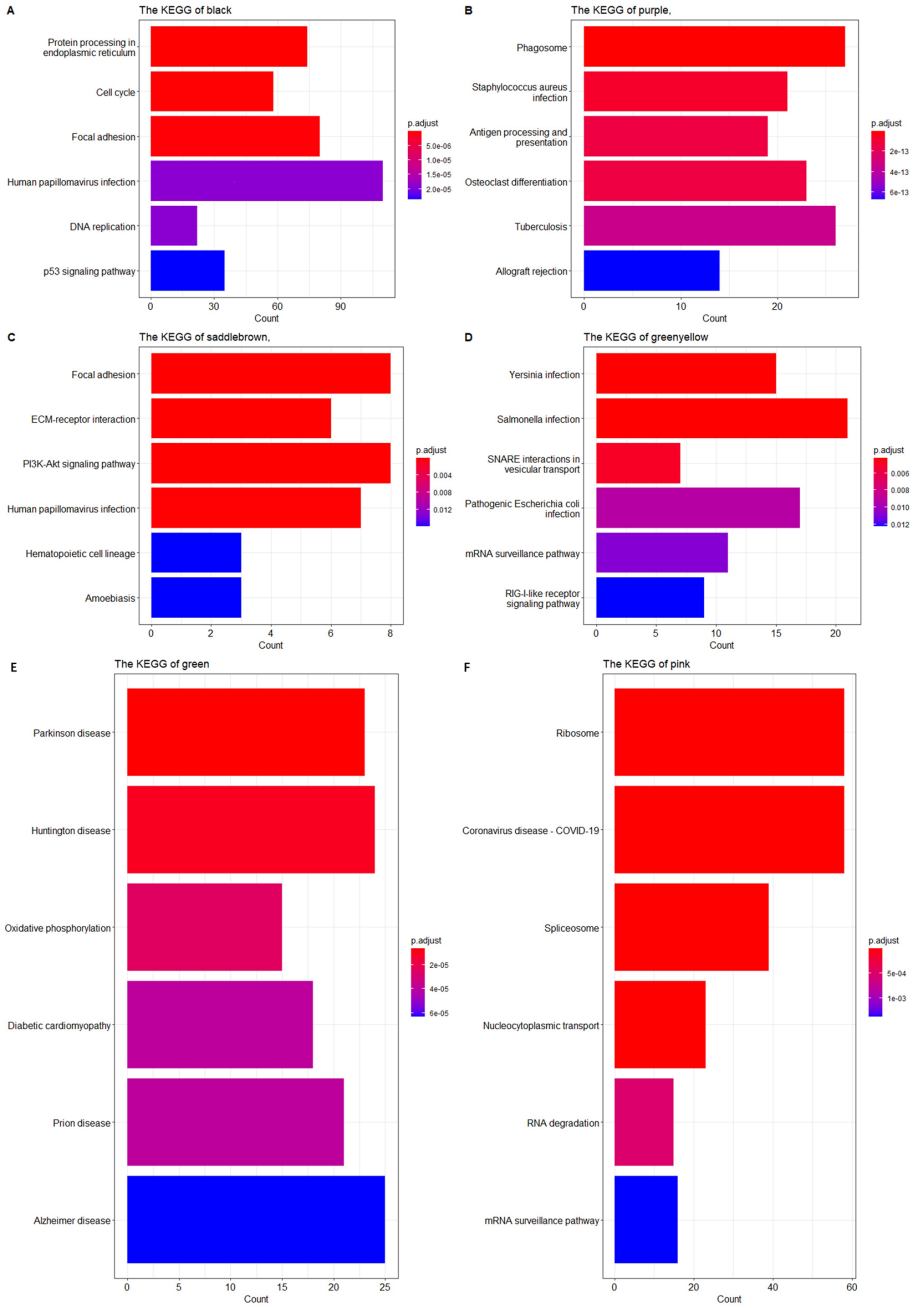
The enriched KEGG terms of the genes for several modules. (**A**–**F**) Shows enriched KEGG terms for black, purple, saddlebrown, greenyellow, green and pink modules respectively.

The black module exhibits a significantly positive correlation, where the enriched Gene Ontology (GO) terms are primarily associated with the cell cycle, as seen in Fig. 4A. Additionally, the enriched KEGG pathway within the black module includes the p53 signaling pathway (Fig. 5A). For the purple module, the enriched GO: Biological Processes (BP) and KEGG terms prominently include antigen processing and presentation (Figs. 4B and 5B). The saddlebrown module, as depicted in chart Fig. 5C, exhibits an enrichment in pathways including focal adhesion, ECM-receptor interaction, and PI3K-Akt signaling pathway. Figure 5D represents the greenyellow module, highlighting infections such as Yersinia and Salmonella infections, and the SNARE interactions in vesicular transport pathway, with statistical significance denoted by varying color intensities. In the green module, Fig. 5E delineates neurological disease pathways, including Parkinson's disease, Huntington's disease, and Alzheimer's disease, all of which have varying degrees of gene involvement and statistical significance. Lastly, the pink module detailed in Fig. 5F includes pathways such as the ribosome, Coronavirus disease—COVID-19, and RNA degradation, again represented by the count of genes and adjusted p-values, indicating their significance in the module's correlation.

Among the modules negatively correlated with Grade4, the enriched GO terms in darkgrey module are mainly related to ensheathment of neurons, axon ensheathment , myelination, glial cell development and differentiation. For grey60 module, the enriched GO:BP terms include gamma-aminobutyric acid secretion, transport and signaling pathway, neuron-glial cell signaling. The genes in turquoise module mainly participated in axonogenesis and regulation of neuron projection development. The enriched Go terms in green module are mainly related to cellular respiration such as respiratory electron transport chain.

Our functional enrichment analysis delineated significant biological processes and pathways associated with Grade 4 GBM. Specifically, modules identified in black and purple showed enrichment in cell cycle regulation and antigen processing and presentation pathways, highlighting their potential roles in tumor proliferation and immune response mechanisms. Notably, the saddlebrown module's enrichment in glomerular development and ECM-receptor interaction pathways suggests a link to tumor microenvironmental dynamics and possible implications for cell adhesion processes. Moreover, modules depicted in darkgrey and grey60 underscore neuronal-related processes, such as myelination and GABAergic signaling, which may reflect the tumor's impact on neural function. The turquoise module's association with axonogenesis underscores the aggressive nature of GBM, implicating alterations in neuronal development and signaling pathways.

### PPI network construction and screening of hub genes

The higher the correlation between genes and specific external traits, the larger their kIM value. These genes are selected and imported into STRING to establish PPI network, which is then visualized in cytoscape^10,11^. The cytohubba plugin is used to calculate degree of every gene in PPI^12^. Some functionally related proteins in a protein network tend to form complexes, meaning that such neighbor component interact more easily than other proteins in the network. MCODE plugin is used to find the clusters in PPI^13^.

**Figure 6:**
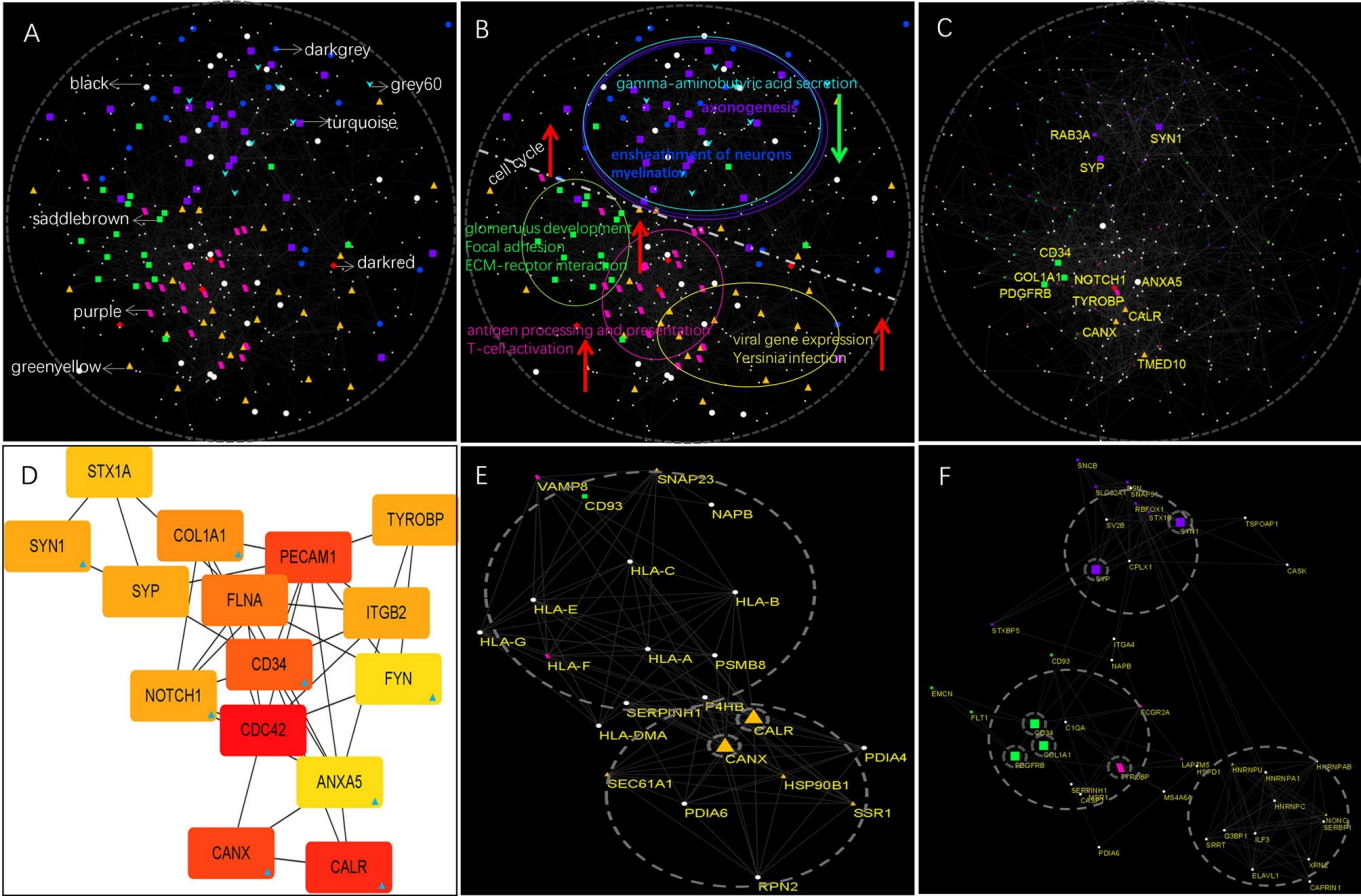
Distribution of genes in modules identified by WGCNA in the spatial protein–protein interaction network. A map of network was generated in Cytoscape. (**A**) The protein–protein interaction network was constructed by connecting all the genes in our study. All the genes from 8 modules were colored in different colors as shown. (**B**) Different modules are marked with ellipses of the same color as the genes in this module. Red upward arrows indicate positive correlation with GBM, and green downward arrows indicate negative correlation with GBM. The modules negatively correlated with GBM are mainly related to neuronal development and function, and are located in the opposite position of positively correlated modules. (**C**) The distribution of hub genes in PPI network generated by cytohubba plugin. (**D**) The interaction of hub genes. (**E**,**F**) The distribution of hub genes in MCODE components.

Edge-weighted spring embedded layout of co-expression network is shown in Fig. 6A. The modules corresponding to the relevant colors are marked on the figure. It is obvious that the genes of each module are relatively clustered in a specific region in the co-expression network. In order to clearly visualize this feature, we annotate it in Fig. 6B. The red arrow indicates the modules positively correlated with GBM, while the green arrow indicates a negative correlation. If a diameter is delimited, it can be found that the positively and negatively correlated modules are separated into two semicircles, and the cell cycle is evenly distributed throughout the co-expression network. The cytoHubba plugin was used to calculate degree of every gene in the network. High-scoring genes include CDC42, PECAM1, CALR, CANX and FLYN etc. (Fig. 6D). Hub genes belonging to relevant modules are shown in the Fig. 6C.

MCODE analysis demonstrated that the first two significant clusters contained the eight hub genes are shown in Fig. 6E,F.

### The ROC analysis

**Figure 7:**
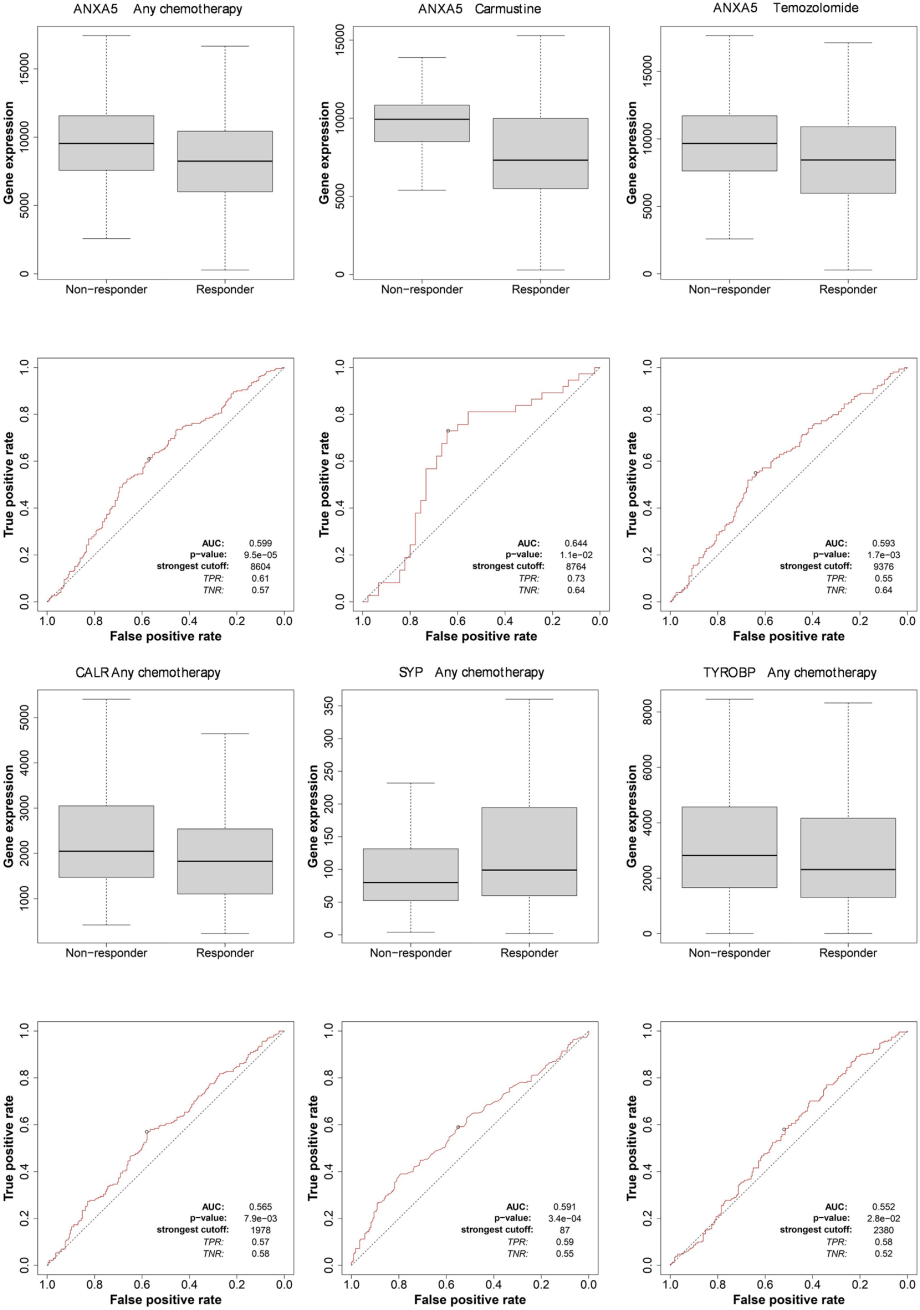
ROC curves and box-plots of hub genes in GBM.

We analyzed four hub genes including ANXA5, CALR, SYP, TYROBP for any chemotherapy in GBM patients. The ROC plotters of the genes with strong correlation to resistance especially chemotherapy (any) in all samples (n = 454) were shown in Fig. 7. We find that ANXA5 has relatively high AUC and low *p*-value (p = 9.5e−5, AUC = 0.599). In order to find the relation between ANXA5 expression and specific drugs, the ROC analysis was performed including carmustine (*p* = 1.1e−03, AUC = 0.644) and temozolomide (*p* = 1.7e−3, AUC = 0.593). It can be speculated that these genes may be the targets of any chemotherapy, especially ANXA5 may be the therapeutic target of anthracycline.

## Discussion

GBM is a malignant tumor that originates in the central nervous system. By studying the molecular mechanisms, gene expression, and signaling pathways of GBM, we can discover new therapeutic targets, thereby developing more effective treatment strategies. This has significant implications for our understanding of the biological characteristics of this disease, improving patient outcomes and quality of life.

The main objective of this study was to gain molecular insights into GBM by constructing a gene co-expression network to identify and predict key genes and signaling pathways associated with GBM. Significantly altered modules were correlated with different stages of the tumor. Genes within the same module were performed GO and KEGG enrichment analysis to obtain significantly altered physiological processes and signaling pathways. Finally, we constructed a protein–protein interaction network using Cytoscape to analyze the correlation between genes in different modules throughout the network.

The positively correlated modules—saddlebrown, black, and purple—manifest associations with the aggressive nature of high-grade GBM. Particularly, the enrichment of cell cycle and immune response genes in these modules aligns with the canonical understanding of oncogenesis where dysregulated cell proliferation and evasion of immune surveillance are pivotal. The statistical robustness of these correlations, as evidenced by the black module's significant p-value, attests to the potential of these genes in driving the malignant phenotype.

Conversely, the negative correlation of modules such as darkgrey, grey60, turquoise, and darkred with high-grade GBM invokes a different facet of the tumor biology—impairment of normal brain functions, such as neuronal development and myelination. The attenuated expression of these genes in higher-grade GBM could suggest a loss of the neuronal identity of glial cells as they acquire a more neoplastic character. These findings may expand our understanding of GBM pathophysiology beyond mere proliferation, encompassing the aberrations in normal brain cell function.

The modules including saddlebrown, black, purple, darkgrey, grey60 and turquoise modules changed significantly at all brain tumor tissues. Several modules, including saddlebrown, black and purple, were significantly positively correlated with GBM. They mainly involved physiological processes such as cell cycle, glomerulus development, focal adhesion, antigen processing and presentation. As we all know, the cell cycle is at the heart of cancer. Once the regulatory process malfunctions, uncontrolled cell proliferation occurs. It can be seen that cell cycle changes occurred early in brain tumor. Some promising candidate drugs for treating GBM, such as benzimidazuoles, may inhibit tumor growth by regulating the cell cycle and epithelial–mesenchymal transition (EMT)^14,15^.

If we artificially divide the differential gene interaction network diagram into two semicircles, it will be obvious that the modules positively correlated with GBM are almost all distributed in the same semicircle (Fig. 6B), while the modules negatively correlated with GBM are almost all located in the opposite semicircle, which should not be a coincidence. We may discover some interesting patterns. Firstly, we can observe that there may be differences in node density between the two semicircles. In one semicircle, there may be more nodes clustered together, while in the other semicircle, nodes may be more dispersed. This difference may be due to different interaction strengths between different genes. Additionally, we divide the differential gene interaction network map into several different color regions to distinguish different modules. This visualization method can help us better understand the mutual relationships and direction of action between genes, and discover potential biological function modules. The negative modules include biological processes related to neuronal development and function including of neurons, axon ensheathment, myelination, glial cell development and differentiation axonogenesis, and regulation of neuron projection development. Recent studies suggest that improving myelin regeneration may be a promising therapeutic strategy for fatal glioblastoma. The findings are consistent with our research^15–17^. This indicates that demyelination or myelin regeneration disorders play a crucial role in the occurrence and progression of glioblastoma multiforme.

There is limited research on the SYP gene, which may be responsible for encoding a structural protein that binds to cholesterol. This protein can organize other membrane components or guide vesicles to the plasma membrane. Its mutations may be related to cognitive impairment and may also involve the regulation of short-term and long-term synaptic plasticity. We speculate that the SYP gene may be involved in the decreased myelin function that occurs in glioblastoma development. From the ROC analysis, it is likely to be a promising target for chemotherapy of glioblastoma.

Annexin A5 (ANXA5) also named as placental anticoagulant protein I, thromboplastin inhibitor V, endonexin II, calphobindin I and lipocortin V, is a calcium-dependent phospholipid-binding protein. Recent research findings indicate that overexpressed ANXA5 can inhibit the proliferation and metastasis of cervical cancer cells, thus exerting its anti-cancer gene function^18,19^. Anxa5 could be a diagnostic, prognostic and therapeutic significance in cancer^20^. Studies on colorectal adenocarcinoma have shown that the expression of ANXA5 is associated with higher tumor stage^21^. From the ROC results, it can be seen that ANXA5 is likely to be a promising target for chemotherapy of glioblastoma.

Several studies suggest that TYROBP (Transmembrane Immune Signaling Adaptor TYROBP) may be an ideal marker for various tumors. Moreover, the higher expression of TYROBP correlates with a poor prognosis in Kidney renal clear cell carcinoma patients^22^. In our study, it is likely to be a promising target for chemotherapy of glioblastoma.

In summary, our study has identified several biological processes strongly linked to GBM when compared to both healthy brain tissue and other grades of brain tumors. Additionally, we conducted a screening of potential key genes and assessed their potential as chemotherapy targets, with the anticipation that they could serve as therapeutic markers for GBM in the future. This research establishes a crucial theoretical foundation for advancing our understanding of GBM’s occurrence and treatment.

## Materials and methods

### Data processing

In order to explore the important genes, physiological processes and signaling pathways related to GBM pathogenesis, The dataset (GSE:4290) employed in this study was acquired from the NCBI Gene Expression Omnibus (GEO), comprising a total of 176 samples as documented by Sun et al.^23^. These samples were classified into four groups, specifically normal (non-tumor), Grade 2 (astrocytomas), Grade 3 (oligodendrogliomas), and Grade 4 (glioblastomas).

### Construction of weighted gene co-expression network and identification of modules related to external traits

WGCNA is an unsupervised analysis method designed to group genes based on their expression profiles^24,25^. In this study, we employed WGCNA to construct a gene co-expression network for GBM and to identify gene modules associated with various GBM grades. WGCNA is a gene clustering method based on the similarity of expression patterns, allowing us to capture and analyze the modular structure within complex gene expression data. Initially, using gene expression data from all samples, we constructed a weighted network and selected an appropriate soft threshold power by analyzing the network's scale-free topology to ensure that the network maintained scale-free properties. Through dynamic tree cutting methods, we identified a series of gene modules and further analyzed the correlation between these modules and the clinical features of GBM. Functional enrichment analysis was then performed on the genes within each module to identify those associated with specific biological processes and signaling pathways. Furthermore, we assessed the correlation between modules and clinical traits of GBM to identify potential biomarkers related to disease progression. The WGCNA package, available for free, is a valuable tool for identifying modules of highly correlated genes^3^.

### Correlation of WGCNA modules with clinical traits

In our approach to correlate the modules identified via Weighted Gene Co-expression Network Analysis (WGCNA) with clinical traits of glioblastoma multiforme (GBM), we analyzed the association between module eigengenes (MEs) and GBM grades. MEs are the first principal component of a module and capture the majority of variation within the module's gene expression profiles. We correlated these MEs with external traits, such as tumor grades, using Pearson correlation to ascertain the statistical significance of their association with GBM severity. For modules showing notable correlations, such as those in saddlebrown, black, purple, and greenyellow, we observed a positive correlation with higher GBM grades, indicating their potential involvement in more aggressive tumor phenotypes. The black module, for example, showed a highly significant correlation (p-value of 4e−22) with Grade 4 GBM. Conversely, modules like darkgrey, grey60, turquoise, and darkred displayed negative associations with Grade 4 tumors. By assigning clinical relevance to these gene co-expression modules, we aim to pinpoint molecular signatures that could have implications for prognosis and therapy. This methodology ensures that the connections drawn from gene expression patterns to clinical observations are statistically robust and biologically interpretable, providing a substantive foundation for subsequent analyses and potential clinical applications.

### Functional enrichment analysis

The Clusterprofiler package is used to perform functional enrichment analyses for biological processes (GO:BP) and Kyoto Encyclopedia of Genes and Genomes (KEGG) analyses to obtain enrichment results for genes in each module identified by WGCNA^26^. The cutoff value for p-value and q-value was set to 0.05 and 0.2 respectively in our analysis.

### PPI network construction and hub gene identification

The identification of hub genes within the co-expression modules derived from WGCNA was based on their intramodular connectivity measure, known as the kIM value. A higher kIM value indicates a stronger connection or correlation of a gene with the particular traits of interest, such as the severity of GBM. These hub genes are pivotal within their respective modules, potentially exerting significant influence on the module's biological function. For the construction of the PPI network, we imported these hub genes into the STRING database, an extensive resource for known and predicted protein–protein interactions. The resulting networks were visualized in Cytoscape, which facilitates the analysis of biological pathways and networks on a graphical interface. The PPI network construction was intended to hypothesize how these hub genes might interact at the protein level, providing a proteomic perspective to the transcriptomic findings. To identify potential protein complexes within the PPI network, which may correspond to functional clusters in GBM, we utilized the MCODE plugin in Cytoscape. This tool helps to find densely connected regions, or clusters, within the PPI network by focusing on the neighborhood density and assigning a score based on the connectivity of the network components.

### Gene expression and therapy response correlation analysis

To investigate the correlation between gene expression and chemotherapy response, we performed a ROC analysis on genes identified as central within their respective co-expression modules from WGCNA. The hub genes were selected for their high intramodular connectivity and potential relevance to GBM pathogenesis. Using the ROC Plotter (https://www.rocplot.org/)^27^, we quantitatively assessed each gene's predictive power regarding the patient response to standard chemotherapeutic treatments. This method aimed to identify potential biomarkers for gauging treatment efficacy, with the ultimate goal of informing personalized therapy strategies for GBM patients.

## Data Availability

The data that support the findings of this study are openly available in GEO at https://www.ncbi.nlm.nih.gov/geo/query/acc.cgi?acc=GSE4290, reference number^23^.
